# Engineering fluorinated-cation containing inverted perovskite solar cells with an efficiency of >21% and improved stability towards humidity

**DOI:** 10.1038/s41467-020-20272-3

**Published:** 2021-01-04

**Authors:** Xiao Wang, Kasparas Rakstys, Kevin Jack, Hui Jin, Jonathan Lai, Hui Li, Chandana Sampath Kumara Ranasinghe, Jaber Saghaei, Guanran Zhang, Paul L. Burn, Ian R. Gentle, Paul E. Shaw

**Affiliations:** 1 Centre for Organic Photonics & Electronics, The University of Queensland, Brisbane, QLD 4072 Australia; 2 Centre for Microscopy and Microanalysis, The University of Queensland, Brisbane, QLD 4072 Australia

**Keywords:** Devices for energy harvesting, Electronic properties and materials

## Abstract

Efficient and stable perovskite solar cells with a simple active layer are desirable for manufacturing. Three-dimensional perovskite solar cells are most efficient but need to have improved environmental stability. Inclusion of larger ammonium salts has led to a trade-off between improved stability and efficiency, which is attributed to the perovskite films containing a two-dimensional component. Here, we show that addition of 0.3 mole percent of a fluorinated lead salt into the three-dimensional methylammonium lead iodide perovskite enables low temperature fabrication of simple inverted solar cells with a maximum power conversion efficiency of 21.1%. The perovskite layer has no detectable two-dimensional component at salt concentrations of up to 5 mole percent. The high concentration of fluorinated material found at the film-air interface provides greater hydrophobicity, increased size and orientation of the surface perovskite crystals, and unencapsulated devices with increased stability to high humidity.

## Abstract (web summary)

Efficient and stable perovskite solar cells with simple active layers are desirable for manufacturing, yet formation of a two-dimensional component in the perovskite film compromises the performance. Here, the authors report low temperature fabrication of highly efficient and stable inverted solar cells by adding a fluorinated lead salt.

## Introduction

The power conversion efficiencies (PCEs) of hybrid three-dimensional (3D) organic–inorganic halide perovskite solar cells (PSCs) based on the standard *n*−*i*−*p* architecture have shown remarkable improvement, with the PCEs of cells based on an inverted structure (*p*–*i*–*n*) generally being lower when containing similar active layers. The improvement in efficiency in both cases has been achieved by manipulation of the constituents within the perovskite active layer leading to materials with high absorption coefficients, long carrier diffusion lengths, and small exciton-binding energies^1–3^. Combining the optimized 3D-perovskite layer with a standard *n*–*i*–*p* device architecture has led to a certified PCE of 25.2%^4^. The very high-efficiency 3D-perovskite solar cells have generally relied on increasingly complex perovskite mixtures (multi-cations and anions), which is an issue for reliable manufacturing. Furthermore, the long-term stability of devices containing 3D perovskites against moisture, heat, and light is still a concern for commercial application of the technology. Therefore, significant effort is now being put towards improving the device stability without sacrificing the efficiency^5,6^.

Two-dimensional (2D) Ruddlesden–Popper (RP) perovskites with a general formula of (RNH_3_)_2_(A)_*n*−1_B_*n*_X_3*n*+1_ have been proposed as a method to improve long-term durability. The increased stability has been attributed to the hydrophobicity and thermal stability of the 2D-perovskite arising from incorporation of large organic spacer cations^7^. Alongside the enhanced stability there has been steady improvement in the efficiencies of 2D-perovskite solar cells with PCEs of up to 18.2% being reported for inverted (*p*–*i*–*n*) devices^8^. However, with 2D-perovskite solar cells, there might ultimately be a trade-off between stability and efficiency with the latter being affected by quantum confinement of the excited state, and a lower carrier mobility and narrower absorption window relative to a range of 3D materials^9–11^. In this sense, mixed 2D/3D hybrid-perovskite materials, i.e., materials that have both 2D and 3D components, provide an attractive conceptual route to combine the advantages of enhanced stability of 2D-perovskites with the strong light absorption and good charge carrier transport properties of a 3D-perovskite active layer^12,13^. However, although in some cases it is clear from the data that there are 2D and 3D crystal types in the active layer in other cases, a 2D/3D structure is assumed simply on the basis that a large large organic cation was added to the processing solution. There is also some confusion in the terminology used with 2D/3D potentially corresponding to simply the large organic cation used, the structure of the bulk of the film, or a bilayer structure with the 2D component on top. Whether or not a 2D/3D-perovskite combination exists and performs well is strongly dependent on the choice of the large organic cation and the design criteria are not yet fully understood. Currently, *n*-butylammonium and phenylethylammonium (PEA) have been the most widely studied large organic cations. They have been reported to impart a 2D/3D structure with improved ambient stability and devices containing them have had PCEs of up to 20%^14,15^. However, the hunt is now on for new organic spacers to achieve the concept of stable 2D/3D-perovskite solar cells. For example, Grancini et al.^16^ reported an ultra-stable (1 year) 2D/3D (5-ammoniumvaleric acid iodide)_2_PbI_4_/MAPbI_3_ perovskite junction with a PCE of 14.6% in standard *n*–*i*–*p* mesoporous solar cells. By introducing the 2D inducing 2,2-(ethylenedioxy)bis(ethylammonium)lead iodide microcrystals into a multication 3D perovskite, Li et al.^17^ realized *n*–*i*–*p* devices with a high steady-state efficiency of 19.7% and encapsulated modules that retained 90% of the initial PCE of 11.6% after 3000 h in air. Zhou et al.^18^ have reported highly efficient, stable 2D/3D hybrid PSCs through incorporation of thiophenemethylammonium iodide spacer cations into a formamidinium/methylammonium-based 3D perovskite, with the optimized device having a PCE of 21.5% in an *n*–*i*–*p* structure. Very recently, fluorinated organic spacer cations have been applied in mesoporous-based *n*–*i*–*p* 2D/3D-perovskite solar cells^14,19^. The choice of the fluorinated spacer cations was reported to be due to their hydrophobic character and superior stability. The highest PCE reported thus far for a 2D/3D device is 22.1% with the structure FTO/titanium dioxide/perovskite/spiro-MeoTAD/Au/antireflective coating^20^. The active layer in this latter device was a bilayer, with the fluorinated ammonium salt dip-coated onto the formamidinium-based perovskite layer followed by an extended thermal treatment. We note that the impressive performance was achieved with an optimized antireflection coating.

Perovskite solar cells historically grew out of research on dye sensitized solar cells and hence many of the developments in materials and record efficiencies have been based on the standard *n*–*i*–*p* architecture using a metal oxide-based electron extraction layer. However, inverted (*p*–*i*–*n*) perovskite solar cells are also attracting attention due to their potential for simple, low-temperature preparation processes, reduced hysteresis, and improved long-term stability under ultraviolet (UV) light^21–23^. There have been few reports of 2D/3D PSCs using the inverted configuration, with most having PCEs lower than their *n*–*i*–*p* device counterparts^15,24^. A complex multicomponent perovskite [(PEA_0.25_F5PEA_0.75_)_2_PbSCN_2x_I_4-2x_]_0.005_[(FA_0.64_MA_0.20_Cs_0.15_)Pb_0.99_(I_0.79_Br_0.2_)_3_] containing a small amount of a fluorinated ammonium cation has been recently reported to have a maximum PCE of 21.10% in an inverted device architecture^25^. However, it is important to note that the efficiency was also achieved with the use of an antireflection coating. Furthermore, although the active film was formed in a single step there was insufficient evidence provided to determine the location of the fluorinated ammonium cation in the film and the maximum amount fluorinated additive incorporated was 1%.

In this work, we incorporate 2-(2,3,4,5,6-pentafluorophenyl)ethylammonium iodide (FEAI)^26^ into the processing solution normally used for the formation of the 3D methylammonium lead iodide (MAPbI_3_) and demonstrate simple perovskite-based inverted solar cells with a champion efficiency of 21.1%. We demonstrate that small quantities of the additive enhance the performance relative to the neat MAPbI_3_ in terms of PCE and stability to humidity and temperature. We also show that when the FEAI concentration in the processing solution is above a critical level the performance of the cells decreases. Importantly, we report that for the high-efficiency cells there is no evidence for the formation of a 2D-perovskite layer nor well defined large 2D crystals. Rather the efficient photoactive films are composed of MAPbI_3_ crystals that are larger at the surface than the bulk with the fluorinated additive concentrated at the surface.

## Results

### Device performance

**Fig. 1:**
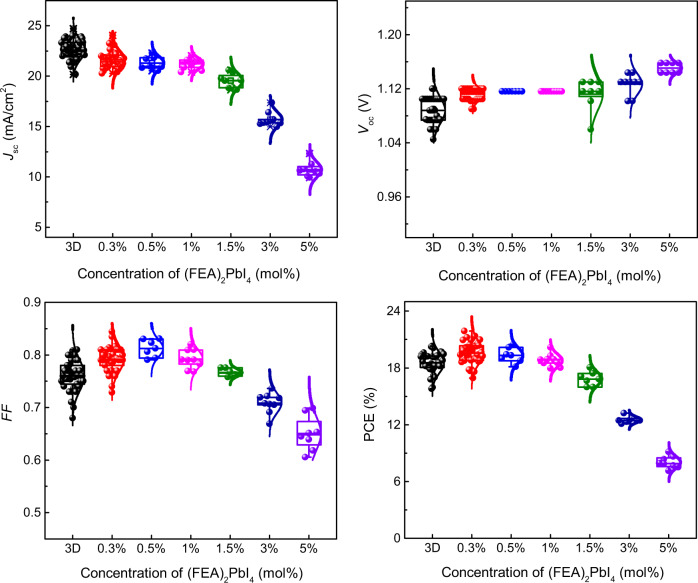
*J*_sc_, *V*_oc_, FF, and PCE distributions for devices comprising MAPbI_3_ and active layers with different mol% of (FEA)_2_PbI_4_. The box is defined by two lines at the 25th percentile and 75th percentile with the line inside the box representing the median (the 50th percentile). The error bars indicate the 5th and 95th percentiles, and the curve overlay represents a normal distribution. At least eight PSCs for each type were tested. Although the MAPbI_3_ devices had on average the highest short-circuit current density (*J*_sc_), addition of a small mol% of (FEA)_2_PbI_4_ to the processing solution led to an increase in the open circuit voltage (*V*_oc_) and fill factor (FF), with an overall increase in the PCE. Although a larger mol% of (FEA)_2_PbI_4_ in the active layer led to a larger *V*_oc_, all the other parameters, and hence the power conversion efficiency (PCE), decreased.

**Fig. 2:**
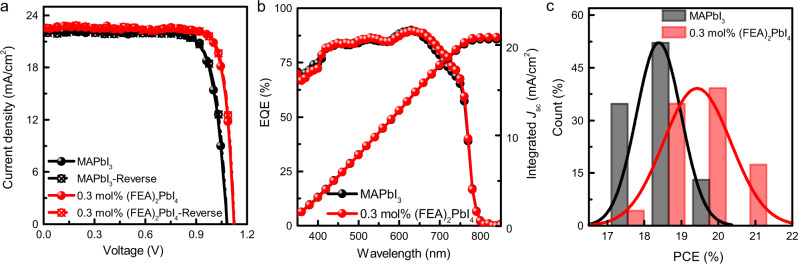
Photovoltaic performance of the neat MAPbI_3_ and 0.3 mol% (FEA)_2_PbI_4_-containing solar cells. **a** Current density–voltage (*J*–*V*) curves of high-performing cells measured in forward and reverse scans (scan rate 200 mV/s) under simulated AM 1.5 G 1 sun irradiation. **b** External Quantum Efficiency (EQE) spectra and integrated short-circuit current density (*J*_sc_) of the corresponding devices. **c** Histogram of the number of cells as a function of power conversion efficiency (PCE) (at least 30 cells for each type of device) fitted with a normal distribution.

The 2D/3D-perovskite active layers are generally formed in one of two ways: either by processing a solution containing the salts that individually would give rise to a 2D or 3D perovskite in the desired ratio or sequentially depositing each type of cation. We note that the high-efficiency *n*–*i*–*p* devices reported by Liu et al.^20^ were prepared by the latter method. In our current work on inverted solar cells, we prepared the perovskite films from solutions containing different mole percent (mol%) of FEAI and methylammonium iodide with the ratio of the salts relative to lead iodide chosen to give the stoichiometry required in principle for the formation of both the 2D [(FEA)_2_PbI_4_] and 3D (MAPbI_3_) perovskites. Thus, the mol% values shown in the figures and used in the discussion are based on the assumption that (FEA)_2_PbI_4_ and MAPbI_3_ are formed quantitatively from their respective salts during processing, although this will be commented on later. The devices had the architecture ITO/PTAA/PFN-P2/perovskite/C_60_/BCP/Cu [where PTAA = poly(bis{4-phenyl}{2,4-bimethylphenyl}amine), PFN-P2 = poly({9,9-bis[3’-({*N*,*N*-dimethyl}-*N*-ethylammonium)propyl]-2,7-fluorene}-*alt*-2,7-{9,9-di-*n*-octylfluorene}), and BCP = bathocuproine], with the performance of the devices containing different mol% of (FEA)_2_PbI_4_ summarized in Fig. 1 and Supplementary Table 1. In addition, the current density-voltage (*J*-*V*) characteristics for high-performing devices of each mol% are shown in Supplementary Fig. 1 and a comparison of devices composed of neat MAPbI_3_ and a 0.3 mol% (FEA)_2_PbI_4_ : MAPbI_3_ are shown in Fig. 2.

In this work, the best MAPbI_3_ inverted devices prepared by a reported method^27^ had a PCE of 19.1%, with an open-circuit voltage (*V*_oc_) of 1.07 V, short-circuit current density (*J*_sc_) of 22.3 mA/cm^2^, and fill factor (FF) of 79%. In common with other reports on inverted perovskite solar cells, the neat MAPbI_3_ device showed little hysteresis between the forward and reverse scans (Fig. 2a). The characteristics of the devices were found to be strongly dependent on the amount of (FEA)_2_PbI_4_ in the film. Addition of (FEA)_2_PbI_4_ led to an increase in the *V*_oc_ with the highest *V*_oc_ of 1.16 V being observed for the 5 mol% (FEA)_2_PbI_4_-containing film. However, although increasing the (FEA)_2_PbI_4_ content improved the *V*_oc_, it was found to be detrimental to the other device parameters with the higher (FEA)_2_PbI_4_ content leading to a significant reduction in *J*_sc_ and FF, and consequently the PCE. We found that the device comprising 0.3 mol% (FEA)_2_PbI_4_ gave the best performance with a maximum PCE of 21.1%, a large *V*_oc_ of 1.12 V, a *J*_sc_ of 22.4 mA/cm^2^, and an FF of 84%. We believe this efficiency is competitive with the highest reported for inverted perovskite solar cells composed of a mixture of 2D and 3D salts in the precursor solution, although with a less complex active layer^25^. Given that the 0.3 mol% (FEA)_2_PbI_4_-containing devices gave the best overall efficiency, we focussed our detailed device study on this blend. An important feature of the high-performing 0.3 mol% (FEA)_2_PbI_4_ devices was that there was negligible current–voltage hysteresis between the forward and reverse scans (Fig. 2a). To show that there was a statistically relevant improvement in performance we prepared and tested over 30 cells of each type [MAPbI_3_ and 0.3 mol% (FEA)_2_PbI_4_] with the histograms shown in Fig. 2c. It can be clearly seen that the 0.3 mol% (FEA)_2_PbI_4_-based devices had an average PCE of 19.5%, which outperformed the neat MAPbI_3_ perovskite-based cells (an average PCE of 18.3%).

### Elucidating the origin of device performance difference

**Fig. 3:**
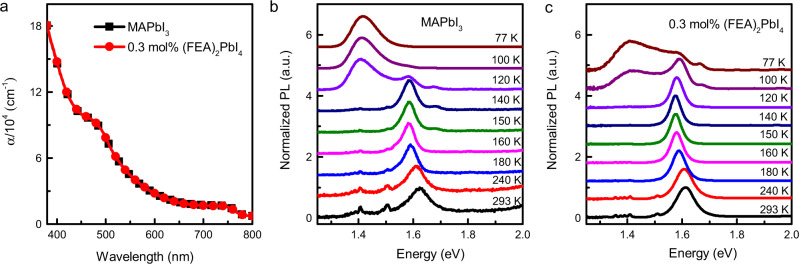
Photophysical properties of the neat MAPbI_3_ and 0.3 mol% (FEA)_2_PbI_4_-containing films. **a** Absorption spectra on glass (*α* = absorption coefficient). **b**, **c** Temperature-dependent (*K* = Kelvin) photoluminescence (PL) spectra (*λ*_exc_ = 550 nm) on fused silica.

To identify the origin of the improved performance of the devices containing 0.3 mol% (FEA)_2_PbI_4_ we first compared the photophysical properties of the photoactive films with a range of different (FEA)_2_PbI_4_ concentrations. The absorption spectra of the neat MAPbI_3_ and 0.3 mol% (FEA)_2_PbI_4_-containing films are overlaid in Fig. 3a [note the absorption spectra are consistent with the External Quantum Efficiency (EQE) response in Fig. 2b], with those of higher (FEA)_2_PbI_4_ content shown in Supplementary Fig. 2 and pictures of the films in Supplementary Fig. 3. It appears at first sight that the absorption spectra of films containing up to 10 mol% (FEA)_2_PbI_4_ were essentially identical to that of the MAPbI_3_ with an absorption onset of around 760 nm^28^, with only the 50 mol% (FEA)_2_PbI_4_-containing film being significantly different. The 50 mol% (FEA)_2_PbI_4_ containing film had distinctive absorbance peaks at around 403, 499, 547, and 569 nm, which were in line with previous reports on 2D/3D perovskite-containing films^29,30^ and could be ascribed to the excitonic absorption of 2D-perovskite phases with *n* = 1–4 layers, respectively. Supplementary Fig. 3 shows how the colour of the films change with increasing (FEA)_2_PbI_4_ content. It can be seen that even though the absorption spectra of the films containing up to 10 mol% of (FEA)_2_PbI_4_ were the same as MAPbI_3_, the surface of the films had an increasingly red hue. The red hue could arise from different reflectivity from the surface of the films due to differences in crystal size and/or orientation (see later discussion on film structure), and/or it might be associated with an increase in the bandgap from quantum confinement effects arising from the 2D component, which becomes increasingly visible at higher concentration^31^. To gain further insight into the effect the (FEA)_2_PbI_4_ content of the absorption of the films, we used Tauc Plots to estimate the bandgaps of the perovskite films. Supplementary Fig. 4 shows that the bandgaps of the films with up to 3 mol% of (FEA)_2_PbI_4_ were within experimental error the same. However, there was a small increase in the absorption edge for higher mol% (FEA)_2_PbI_4_-containing films, suggesting that the latter films might have a slightly greater content of (FEA)_2_(MA)_*n*−1_Pb_*n*_I_3*n*+1_ with large *n* phases^32^. Nevertheless, the fact that there was not a significant difference in the absorption onset or profile for films with <3 mol% (FEA)_2_PbI_4_, which includes the blend that gave rise to the most efficient devices, was indicative of them having only small amounts of, if any, 2D (FEA)_2_(MA)_*n*−1_Pb_*n*_I_3*n*+1_ with small *n* (*n* ≤ 4) or bulk 2D phases throughout the film^33,34^.

Further evidence for the low (FEA)_2_PbI_4_ content films lacking a significant 2D phase component throughout the film came from the photoluminescence (PL) spectra (Supplementary Fig. 5). It was found that the blue shift in the emission normally associated with the formation of a mixed 2D/3D composite, where charge carriers are confined by the large potential barrier caused by the 2D-perovskite component^35,36^, only occurred when the (FEA)_2_PbI_4_ concentration reached >10 mol%. The emission of the 10 mol% (FEA)_2_PbI_4_ content film had a small blue shift, whereas that of the highest concentration [50 mol% of (FEA)_2_PbI_4_] was significantly blue shifted. In addition, emission peaks at <600 nm were also observed for the 50 mol% (FEA)_2_PbI_4_ film, which correlate with the absorption peaks and arise from small n (*n* ≤ 4) 2D phases in the film^36^. It should be noted that the relative PL intensity (Supplementary Fig. 6) and PL decay lifetime (Supplementary Fig. 7 and Supplementary Table 2) both increased with increasing (FEA)_2_PbI_4_ content in the film, which would be consistent with changes in the structure of the film leading to exciton confinement and/or grain boundary defect passivation. The PL decay profiles for the neat MAPbI_3_ and the best-performing 0.3 mol% (FEA)_2_PbI_4_-containing films were essentially the same and could be fitted to a biexponential with a relatively fast component, closely related to recombination induced by defects or impurities, and a slower component arising from radiative recombination occurring in the bulk perovskite^37–39^.

In a final part of the study on the photophysical properties of the films, we measured the temperature dependence of the PL (Fig. 3). For the neat MAPbI_3_ film, we observed the normal change in the PL spectrum at around 120 K, which is due to the phase transition from the tetragonal phase into an orthorhombic phase^40,41^. When the temperature was decreased to 100 K and below, the high-energy emission peak disappears, whereas the low-energy emission intensity increases. Previous studies report that this low-energy emission is due to localized trap-mediated carrier recombination in the perovskite film^42–44^. Adding 0.3 mol% of (FEA)_2_PbI_4_ suppressed the temperature at which the MAPbI_3_ phase transition occurred and at 77 K there was still a clear component relating to the tetragonal phase. When the concentration of the (FEA)_2_PbI_4_ was increased tenfold (3 mol%), similar suppression of the phase transfer with decreasing temperature was observed (Supplementary Fig. 8). In conclusion, in the context of the device performance, analysis of the optical properties of the films showed that the neat MAPbI_3_ and films containing 0.3 mol% of (FEA)_2_PbI_4_ had similar photophysical properties, and hence there must be an alternative reason for the improvement in the device performance. Furthermore, it was found that up to a concentration of (FEA)_2_PbI_4_ of 10 mol% there was little evidence for a significant concentration of the classically described mixed 2D/3D composite throughout the films.

**Fig. 4:**
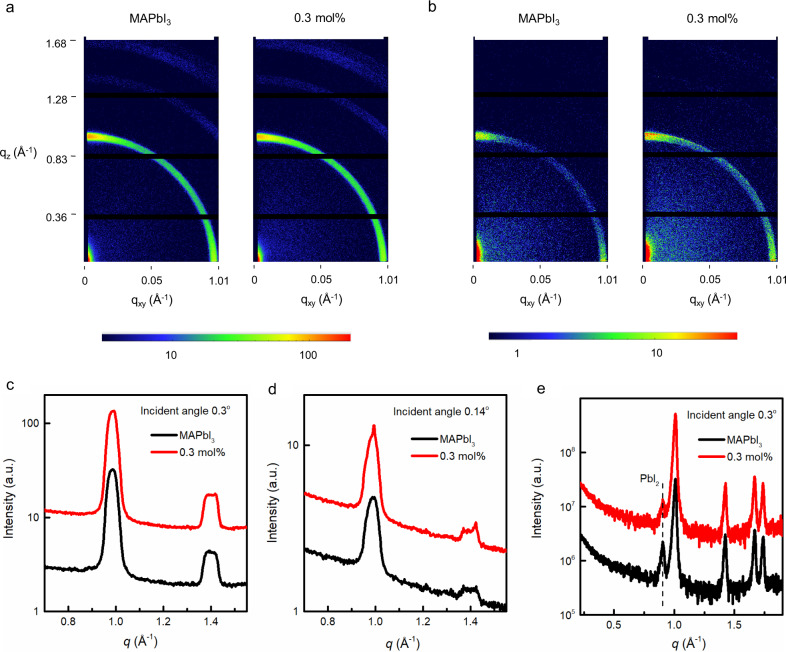
2D GI-WAXS measurements and out-of-plane, high-resolution GI-XRD patterns of the thin films. The 2D GI-WAXS measurements with an incident beam of **a** 0.3° and **b** 0.14° for the neat MAPbI_3_ and 0.3 mol% (FEA)_2_PbI_4_-containing films. Azimuthally averaged GI-WAXS profiles of the neat MAPbI_3_ and 0.3 mol% (FEA)_2_PbI_4_-containing films collected at an incidence angle of **c** 0.3° and **d** 0.14°. **e** Out-of-plane, high-resolution GI-XRD patterns of thin films collected at an incidence angle of 0.3° (note the data have been offset for clarity). *q* = momentum transfer, *q*_*z*_ and *q*_*xy*_ = the components of the scattering vector (*q*) in the direction of the surface normal (*z*) and transverse to it (*xy*).

In the next part of the study, we investigated the structural properties of the materials. The 2D grazing incidence wide-angle X-ray scattering (GI-WAXS) patterns and the corresponding one-dimensional (1D) scattering profiles of the neat MAPbI_3_ and films containing up to 10 mol% of (FEA)_2_PbI_4_ are shown in Fig. 4 and Supplementary Fig. 9. The 1D scattering profiles were obtained from the measured 2D scattering data by radial averaging through the azimuthal angles of 0° to 180°. The measurements are shown at two angles of the incident beam, 0.14° and 0.3°, which are below and above the critical angle of crystalline MAPbI_3_ (ca. 0.26°). At the lower angle, the observed scattering originates from the top few nanometres of the film while at the higher angle the beam probes tens of nanometres into the film providing information on the bulk structure. The predominant feature, which was observed in all of the 2D diffraction patterns is a broad ring at a momentum transfer (*Q*) of ca. 1 Å^−1^ [*q* = 4**sin**(*θ*/2)/*λ*, where *θ* is the angle of the diffraction and *λ* is the wavelength of the incident X-ray beam]. This broad feature can be assigned to the overlapping diffraction from the (002) and (110) planes associated with the tetragonal crystal structure of MAPbI_3_. That is, the films containing up to 10 mol% of (FEA)_2_PbI_4_ comprised MAPbI_3_ crystals. Focussing initially on the measurements with the deeper penetration (higher angle), the overall breadth of the peak (see for example Supplementary Fig. 9c) and the distribution in an arc with an increased intensity close to 0° and 90° (see Supplementary Fig. 9a) are indicative of the individual crystallites being nanosized or disordered and having weak preferential orientation such that the (110) and (002) planes are either parallel or perpendicular with the surface. In general, there was little change in the structure of the material with increasing (FEA)_2_PbI_4_ concentration, except at the highest concentration studied, 10 mol% where there appeared to be some increase in the breadth and changes in the radial distribution of this peak.

In contrast, the GI-WAXS measurements made from the surface of the film (Fig. 4b, d and Supplementary Fig. 9b, d) show both narrowing of the peaks and a higher degree of preferential alignment, i.e., texturing, of the crystallites. This is particularly noticeable for the 10 mol% (FEA)_2_PbI_4_-containing film, as evidenced by the discrete spots along the rings (Supplementary Fig. 9b) and features on the peaks of the 1D profiles (Supplementary Fig. 9d). These observations are consistent with the MAPbI_3_ crystallites at the surface becoming larger in size with addition of the (FEA)_2_PbI_4_. Moreover, there is also evidence for the (110) planes of the material being more strongly aligned parallel with the surface and this surface ordering increases with concentration up to 10 mol%, although there appeared to be a small decrease seen between 0.3 and 0.5 mol%. The difference in orientation and size of crystals at the film surface could lead to different film reflectivity, which in turn could explain why the surface colours of the film change while the absorption spectra are so similar. In summary, these results clearly show that the structure of the films normal to the substrate changes with different mol% of (FEA)_2_PbI_4_. Importantly, the section of the film that is near the air interface is different from the bulk of the film.

To complete the X-ray studies, we undertook high-resolution out-of-plane Grazing Incidence X-ray diffraction (G-XRD) measured as a function of incidence angle and composition with the patterns shown in Fig. 4e and Supplementary Fig. 9e. The films with different mol% of (FEA)_2_PbI_4_ were found to have a crystal structure that is qualitatively similar to that of the neat MAPbI_3_ and different to what would be expected for the 2D RP perovskite, (FEA)_2_PbI_4_. That is, if pure 2D crystals were present in the films, they were either too small or few to be observable. It is conceptually feasible that individual (FEA)_2_PbI_4_ molecules could coat the surface of the MAPbI_3_ crystals or form an amorphous component within the film. Surface coating of the MAPbI_3_ crystals would lead to a reduction in surface defects and be consistent with the increase in the PL signal with increasing (FEA)_2_PbI_4_ content. We also found that in the neat MAPbI_3_ films there was a very weak peak at ca. 0.9 Å^−1^, which can be ascribed to the residual lead iodide in the as-formed film. With increasing addition of the FEAI in the processing solution, the intensity of this peak decreases and completely disappears when >1 mol% of FEAI was added, indicating that the addition of the FEAI reduced the extant PbI_2_ content in the films. The effect of extant PbI_2_ on the performance of MAPbI_3_ devices is still a matter of debate. There are some reports stating that a small excess of lead iodide can lead to devices that exhibit a higher PCE, and that the presence of higher lead iodide concentration can decrease the material and device storage stability^45,46^. In contrast, it has been found that if the PbI_2_ forms sheets within the perovskite layer then improved stability can be achieved^47^.

To further investigate the perovskite film structure, we carried out Scanning Electron Microscopy (SEM) imaging on the complete devices (Supplementary Fig. 10). We found that when the devices were cut/fractured the copper electrode partially delaminated. Nevertheless, it was possible to identify the separate glass, ITO, PTAA/PFN-P2, perovskite, C_60_/BCP and copper layers. We found that the neat MAPbI_3_ perovskite was not highly textured within the bulk of the layer. As increasing amounts of FEAI were added to the perovskite layer it was found that the apparent average crystal size in the bulk decreased. However, it should be noted that increasing the amount of FEAI made the perovskite layers more prone to degradation under the measurement conditions and hence care had to be taken to avoid the introduction of artefacts. It would be reasonable to conclude that the differences observed in the X-ray data for the surface and bulk crystals within the films were due to the film surface being enriched with fluorinated (FEA)_2_PbI_4_.

**Fig. 5:**
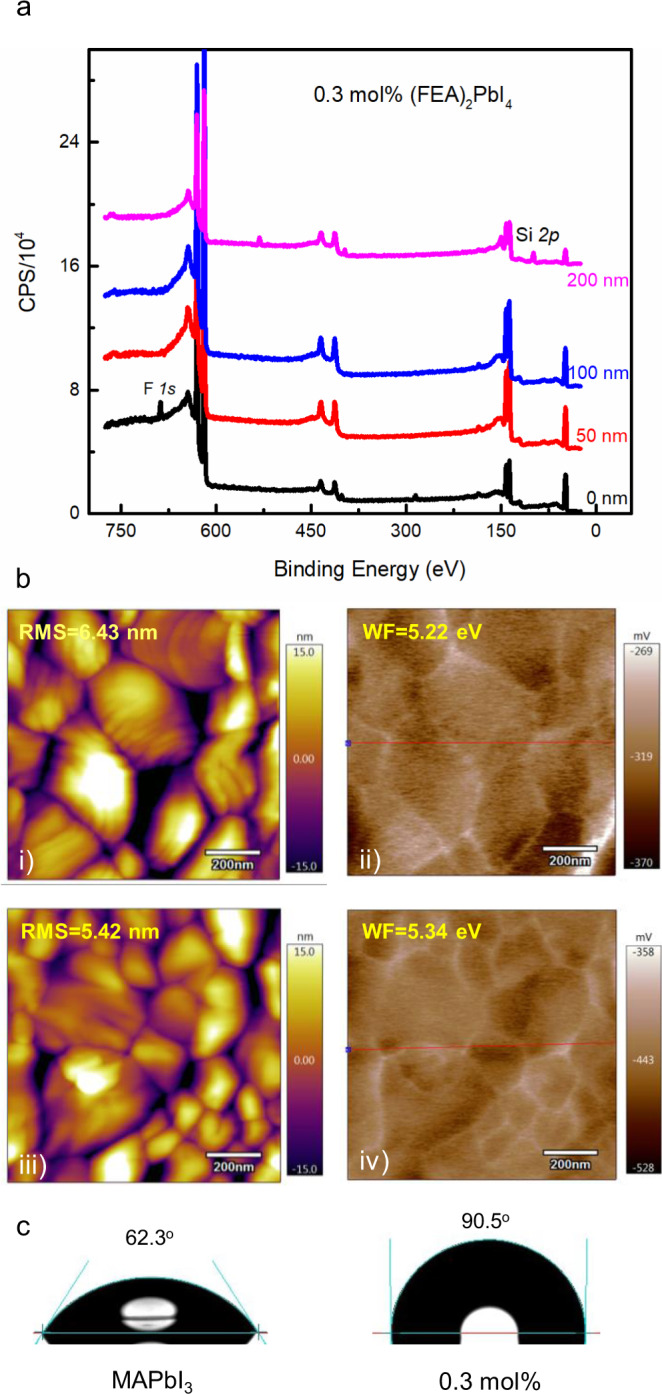
XPS, AFM, SKPM and water contact angle measurements of the thin films. **a** XPS depth profile on a 0.3 mol% (FEA)_2_PbI_4_-containing perovskite film (CPS = counts per second). **b** AFM of the surface of the (i) MAPbI_3_ and (iii) 0.3 mol% (FEA)_2_PbI_4_-containing perovskite films and work function images of (ii) MAPbI_3_ and (iv) 0.3 mol% (FEA)_2_PbI_4_-containing perovskite films obtained from the SKPM measurements (RMS = root mean square roughness and WF = work function). **c** Images of water droplet contact angle measurements of MAPbI_3_ and 0.3 mol% (FEA)_2_PbI_4_-containing films on glass/ITO/PTAA/PFN-P2 substrates.

To study this, we used X-ray photoelectron spectroscopy (XPS) depth profiling of the signal associated with the fluorine atoms to determine where the (FEA)_2_PbI_4_ was located in the film. The 0.3 mol% (FEA)_2_PbI_4_-containing film was found to have a relatively high (19 atom%) concentration of fluorine within 50 nm of the surface with a negligible (<0.7 atom%) in the bulk of the film (Fig. 5a). We note that if the 0.3 mol% (FEA)_2_PbI_4_ was evenly distributed throughout the film then the average fluorine concentration would be approximately 0.4 atom% of fluorine and would be below the detection limit of the XPS measurements. Furthermore, concentration of 19 atom% is a clear indication that the (FEA)_2_PbI_4_ must be at the top surface (50 nm of the 220 nm film). That is, during the solution processing to form the thin film, the (FEA)_2_PbI_4_ concentrates near the air interface. Supplementary Fig. 11 also shows the profiles for 3 mol% and 10 mol% (FEA)_2_PbI_4_-containing perovskite films, and it can be clearly seen that in all cases the concentration of the fluorine atoms was highest near the surface of the film. Furthermore, the absolute concentration of the fluorine atoms was found to increase near the surface with increasing (FEA)_2_PbI_4_ concentration. Also, with increasing (FEA)_2_PbI_4_ concentration, the depth at which the presence of the fluorine atoms was measured was found to increase. These results are also consistent with the increased PL signal seen for the higher (FEA)_2_PbI_4_ content films. That is, with the (FEA)_2_PbI_4_ distributed more deeply within the film a greater degree of MAPbI_3_ crystal surface passivation could occur. We were interested to see whether the phase separation that occurred during the single processing step for MAPbI_3_ was more general and so we carried out a similar set of experiments with the formamidinium lead triiodide, with the results shown in Supplementary Fig. 12. It can be clearly seen that a similar phase separation process occurred, which suggests that the effects observed for the MAPbI_3_ devices might be more general.

A higher (FEA)_2_PbI_4_ content at the surface of the perovskite film would be expected to lead to an increase of the work function due to the electronegativity of the fluorine atoms. We therefore used scanning Kelvin probe microscopy (SKPM) to measure the surface potentials of the films and mapped these onto the surface morphologies from atomic force microscopy (AFM) measurements. Figure 5b shows that the average work function of the 0.3 mol% (FEA)_2_PbI_4_-containing film was greater than that of neat MAPbI_3_ and increasing the concentration caused a further increase in the average work function (Supplementary Fig. 13). By comparing the AFM and SKPM images, we found that the surface potential difference of the films was highest at the grain boundaries, which is consistent with a higher concentration of the (FEA)_2_PbI_4_ being present in between the grains.

### Solar cell stability

**Fig. 6:**
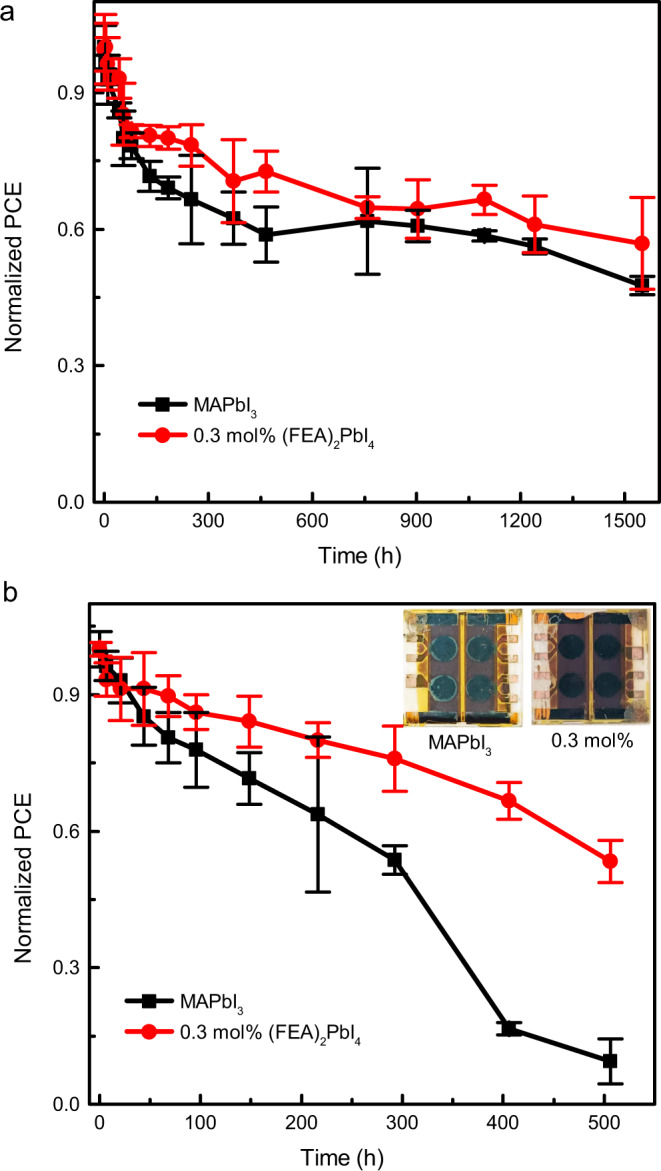
Stability test of the MAPbI_3_ and 0.3 mol% (FEA)_2_PbI_4_-containing devices without encapsulation. **a** Thermal stability test at 85 °C in a nitrogen atmosphere. **b** Humidity test in air with a relative humidity of 80 ± 10% at RT (PCE = power conversion efficiency).

Long-term stability measurements of the optimized MAPbI_3_ and 0.3 mol% (FEA)_2_PbI_4_-containing solar cells were carried out under two different conditions. The unencapsulated cells were either stored in the dark at 85 °C in a nitrogen-filled glovebox or in the dark in air at a relative humidity of 80 ± 10% and measured at periodic intervals (Fig. 6). Under our measurement conditions, both thermally treated devices showed an initial relatively rapid decrease in performance with T80s of around 55 and 183 h for the MAPbI_3_ and 0.3 mol% of (FEA)_2_PbI_4_ devices, respectively (Fig. 6a). However, over a longer time period it was found that the device comprising 0.3 mol% of (FEA)_2_PbI_4_ continued to show slightly higher stability to heat compared to the MAPbI_3_ cells, with T50 values of >1550 and 1450 h, respectively (note the measurements were stopped when one device type reached T50). The decline of the PCE was mainly attributed to the deterioration of the FF (Supplementary Fig. 14). The thermal stability is attributed to the passivation of defects at the grain boundaries by the (FEA)_2_PbI_4_, which is consistent with the results from the SKPM measurements and the PL intensity and PL lifetime measurements. However, the 0.3 mol% (FEA)_2_PbI_4_-containing device showed significant improvement in stability to humidity relative to the MAPbI_3_ cells. The T80 and T50 for the MAPbI_3_ cells were 69 and around 350 h (Fig. 6b), respectively, with the decrease in the PCE mainly caused by a significant decrease in the *J*_sc_ (Supplementary Fig. 15). In contrast, the devices containing the 0.3 mol% of (FEA)_2_PbI_4_ under the same humidity conditions had a T80 of 217 h and T50 was not reached during the measurement time of around 500 h, demonstrating the potential for the strategy to provide improved stability to humidity.

The superior stability towards high humidity of the 0.3 mol% (FEA)_2_PbI_4_-containing devices would be consistent with the surface of the films being more hydrophobic than MAPbI_3_ due to the fluorinated lead salt. Figure 5c shows images of the water contact angle measured for the MAPbI_3_ and 0.3 mol% (FEA)_2_PbI_4_-containing perovskite films on glass/ITO/PTAA/PFN-P2 substrates. It can be seen that the MAPbI_3_ film had a contact angle of 62° with that of the 0.3 mol% (FEA)_2_PbI_4_-containing being 90°. Furthermore, the contact angle increased with the increasing mol% of the (FEA)_2_PbI_4_ (Supplementary Fig. 16), indicating that the perovskite films containing the fluorinated organic cation were more hydrophobic.

We also tested the stability of the devices using maximum power point tracking under simulated AM 1.5 G illumination with the results shown in Supplementary Fig. 17. We found that the PCE of the MAPbI_3_ device decreased rapidly (35% in ≈7 h), which was similar to that previously reported^48^. The burn-in observed for the MAPbI_3_ devices has been ascribed to amorphous regions containing a high density of ionic defects, which when removed through a series of post-fabrication steps can lead to  devices with greater stability^48^. In contrast, the 0.3 mol% (FEA)_2_PbI_4_-based device showed a significantly decreased PCE decay rate under maximum power point tracking conditions. Within the first 10 h, there was only a 22% decrease and after 100 hours the PCE had dropped to 40% of its initial value. The fact that decay was slower for the 0.3 mol% FEAI-containing device could be due to a decrease in ionic defects and increase in crystal ordering at the surface of the perovskite layer induced by the FEAI.

## Discussion

Drawing the results together provides a picture of the perovskite film that gives rise to inverted perovskite solar cells with a hysteresis-free maximum PCE of 21.1% (without the use of an antireflection coating) and improved stability to heat and humidity. Addition of small amounts of FEAI to the processing solution used to form the perovskite led to films in which the (FEA)_2_PbI_4_ vertically phase separated such that it was concentrated near the electron transport interlayer in the devices. At low loading (0.3 mol%) the majority of the (FEA)_2_PbI_4_ was within the first 50 nm of the top of the film. It was found that the (FEA)_2_PbI_4_ did not form a clearly defined 2D-perovskite capping layer or 2D crystals of significant size when mixed with the MAPbI_3_ at concentrations of up to 10 mol%. Instead, at these concentrations the films were composed of MAPbI_3_ with the MAPbI_3_ crystallites at the surface being generally larger in size and having the (110) planes of the material more strongly aligned parallel with the surface. Although the room temperature photophysical properties of the 0.3 mol% (FEA)_2_PbI_4_ content films were essentially identical to the MAPbI_3_, the presence of the small amount of (FEA)_2_PbI_4_ was sufficient to depress the phase transition temperature. Coupling the results from the water contact angle and SKPM measurements with the fact that increasing the (FEA)_2_PbI_4_ content led to an increase in the PL efficiency and lifetime, the results provide a picture where the (FEA)_2_PbI_4_ found at the surface and grain boundaries passivates the MAPbI_3_ crystallite surface defects within the perovskite film. These results show additives that can in principle form 2D RP perovskite films when mixed with a 3D perovskite do not necessarily have to be used at a concentration to form a 2D/3D mixed perovskite film to give devices with good stability and performance. The results show that if there is no FEAI in the active layer composition (pure MAPbI_3_) or there is too much additive (as little as a few percent), then the devices have poorer performance. The addition of 0.3 mol% of FEAI led to perovskite films in which there was no observable 2D phase and devices with the best performance. Thus, the results relax the requirement for 2D phases to be clearly present in the perovskite active layer to overcome the traditional trade-off between the PCE and stability in 2D and 3D PSCs.

## Methods

### Materials

Methylammonium iodide (MAI, >99%) was purchased from GreatCell Solar Limited. Lead iodide (PbI_2_, >98%) was purchased from Tokyo Chemical Industry Co. Ltd. PTAA, PFN-P2, and BCP (>99.5 %) were purchased from Sigma-Aldrich. C_60_ (>99.9%) was purchased from Alfa Aesar. All commercial materials were used as received without any further purification. FEAI was synthesized following a previously reported procedure^49^. The perovskite precursor solution was prepared as follows: for the MAPbI_3_ precursor solution, 1.2 M PbI_2_ and 1.2 M MAI were dissolved in a mixed solvent of γ-butyrolactone (GBL, ≥99%, Sigma-Aldrich) and dimethyl sulfoxide (DMSO, ≥99.9%, Sigma-Aldrich) (7 : 3 v/v) with stirring and heating at 65 °C for 2 h, and then stirring overnight at room temperature. For the mixed mol% (FEA)_2_PbI_4_ precursor solutions, the corresponding amount of MAI was replaced with FEAI. For example, MAPbI_3_ with 0.3 mol% (FEA)_2_PbI_4_ was formed from a precursor solution containing 2.2 mg of FEAI, 171.2 mg of MAI, and 497.9 mg PbI_2_ dissolved in 0.9 mL solvent (GBL:DMSO 7 : 3 v/v). The solution was spin-coated through a two-step process (1000 r.p.m. for 10 s and then 5000 r.p.m. for 80 s). During the second step, toluene (200 µL, 99.9%, Sigma-Aldrich) was dropped after 15 s onto the middle of the rotating substrates. The film was then heated at 100 °C for 30 min. All the films were formed under inert conditions (<1 p.p.m. O_2_; H_2_O) in a nitrogen-filled glovebox.

### Perovskite solar cell fabrication

The PSCs were fabricated on commercial ITO patterned glass electrodes (20 Ω sq^−1^: Xinyan) in a class 1000 clean room. The ITO substrates were sequentially cleaned in an ultrasonic bath with Alconox, Milli-Q water, acetone and 2-propanol for 15 min each. After being blow-dried with nitrogen, the substrates were treated with UV-ozone for 30 min. PTAA in toluene (2.5 mg/mL) was then spin-coated at 6000 r.p.m. for 30 s onto the ITO. The substrates were put onto a hotplate for 10 min at 100 °C. After cooling for 5 min, a thin PFN-P2 (0.5 mg/mL in methanol) layer was spin-coated at 5000 r.p.m. for 20 s onto the PTAA film, to improve the wetting of the perovskite layer. After deposition of the perovskite layer the substrates were then transferred to a high vacuum chamber to deposit 20 nm C_60_ and 7.5 nm of BCP. Finally, 100 nm of Cu was deposited as the cathode. The active area was defined by a shadow mask to be 0.071 cm^2^. Apart from the ITO preparation, all device fabrication steps were performed under inert conditions (<1 p.p.m. O_2_; H_2_O) in a nitrogen-filled glovebox.

### Film characterization

The absorption spectra were measured with a Cary 5000 UV-visible spectrophotometer. The steady-state PL spectra were measured using a Horiba Jobin-Yvon Fluoromax fluorometer at an excitation wavelength of 450 nm. The time-resolved PL decays were measured using a Fluorolog-3 with time-correlated single photon counting capability. The excitation source was a light-emitting diode emitting at 441 nm pulsed at 1 MHz with a pulse width of 1.2 ns and the PL decays were measured at 767 nm. A long-pass filter was used to block any scattered excitation. The instrument response function (IRF) was determined by removing the long-pass filter and measuring the excitation scattered off the sample at 441 nm. The data were fitted with the software application DAS6 (supplied by Jobin-Yvon) using multi-exponential decays convolved with the IRF. Temperature-dependent PL measurements were undertaken on films deposited on glass substrates. The samples were mounted in a cryostat (OptistatDN2) and cooled by liquid nitrogen. The samples were excited at 550 nm with the excitation generated using a 450 W CW Ozone-free Xenon arc lamp. The PL emission from each sample was collected using a HORIBA Fluorolog-3 spectrophotometer with a near IR PMT detector. The film thicknesses were measured using a Dektak 150 Profilometer. The contact angles were measured with a PSS OCA20 (DataPhysics Instruments GmbH) optical contact-measuring system. The surface topography and potential imaging were performed using a Cypher-Asylum Research AFM. The measurements were done in SKPM mode using a Pt-coated cantilever (NSG03 model from NT-MDT) as the conductive probe^42^. The films for these measurements were prepared in the same way as those used for the device fabrication. High-resolution XRD measurements of the films were obtained on a Rigaku Smartlab equipped with a 9 kW Cu rotating anode (operating at 45 kV and 200 mA) and a Rigaku HyPix3000 detector. Out-of-plane GI diffraction data were obtained at an incidence angle 0.3 degrees, using a narrow 0.1 mm-thick × 5 mm-long beam and a 0.115° Soller slit prior to the detector. 2D WAXS measurements were collected on a Xenocs Xeuss 2.0 SAXS instrument. The data were collected using a Cu-Kα (*λ* = 1.542 Å) micro focused tube source operating at 50 kV and 0.6 mA, and a beam size of ca. 0.5 × 0.5 mm. The scattering measurements were collected with the sample under vacuum in GI geometry using a Pilatus 1 M detector at a sample-to-detector distances of 310 mm (calibrated using LaB_6_). Thin films of the samples were deposited onto silicon wafers (1 cm × 1 cm), and the angle of incidence and surface normal were aligned to the beam by iterative half-cut and rocking curve measurements. The XPS profile was collected using a Kratos Axis Ultra XPS Surface Analysis System with a monochromatic Al Kα X-ray source. Elemental depth information was collected using XPS in conjunction with argon-ion bombardment. The argon ion-beam etching was carried out at a beam energy of 4 keV. Each etching step lasted for 3 min, corresponding to an etching thickness of about 50 nm. The atomic percentages are evaluated at each profile step by peak fitting using Avantage software.

### Solar cell characterization

The current–voltage curves were measured under carefully controlled conditions (in a glovebox with O_2_ < 1 p.p.m. and H_2_O < 1 p.p.m.) with a Keithley 2400 source-measurement unit under 1 sun illumination (AM 1.5 G, ~100 mW/cm^2^) with a scan rate of 200 mV/s. The solar simulator (Sun 2000, class AAB Abet Technologies) was calibrated with a National Renewable Energy Laboratory (NREL)-certified standard 2 cm × 2 cm silicon photodiode with a KG5 filter. The cells were excited through a 0.3 cm diameter mask that matched the size of the cells. The mismatch factor between the certified KG5-filtered silicon photodiode standard and the perovskite cells was determined to be 1.09. The reported PCEs are corrected for the mismatch factor. The EQE was measured using a PV Measurements, Inc. QEX7. The QEX7 system was calibrated using an NREL certified photodiode and operated without white-light bias and chopped and locked in the small perturbation limit. Integrated EQE currents were cross-checked for consistency with the white-light short-circuit current. The cells for the EQE measurements were masked and the diameter of the illumination spot size adjusted to the minimum (≈0.3 cm). The optical fibre used for the illumination was manually adjusted to illuminate through the mask aperture. The error in integrated *J*_SC_ from the EQE measurements arising from the alignment process was ≈4%.

### Stability test

For the humidity stability tests, the 3D MAPbI_3_ and 0.3 mol% (FEA)_2_PbI_4_ devices were stored in dark at room temperature without encapsulation. The relativity humidity was maintained at 80 ± 10%. The thermal stability test was conducted by heating non-encapsulated devices in an oven at 85 °C in a nitrogen-filled glovebox (<1 p.p.m. O_2_; H_2_O) in the dark.

### Reporting summary

Further information on research design is available in the Nature Research Reporting Summary linked to this article.

## Supplementary information

Supplementary Information

Reporting Summary

## Data Availability

The data that support the presented plots of this study and other relevant findings are available from the corresponding author on reasonable request.
