# Supplementary material for: Engineering fluorinated-cation containing inverted perovskite solar cells with an efficiency of >21% and improved stability towards humidity
Source: Nat Commun. 2021 Jan 4;12:52. doi: 10.1038/s41467-020-20272-3 (PMC7782759; doi:10.1038/s41467-020-20272-3)
Supplement: Supplementary file 1 — Supplementary Information [file 41467_2020_20272_MOESM1_ESM.pdf]

Engineering fluorinated-cation containing inverted perovskite solar cells with an efficiency of >21% and improved stability towards humidity

Xiao Wang[1], Kasparas Rakstys[1], Kevin Jack[2], Hui Jin[1], Jonathan Lai[1], Hui Li[1], Chandana Sampath Kumara Ranasinghe[1], Jaber Saghaei[1], Guanran Zhang[1], Paul L. Burn[1]*, Ian R. Gentle[1], Paul E. Shaw[1]

[1]Centre for Organic Photonics & Electronics, The University of Queensland, Brisbane, QLD 4072, Australia, [2]Centre for Microscopy and Microanalysis, The University of Queensland, Brisbane, QLD 4072, Australia

* Corresponding author e-mail: p.burn2@uq.edu.au

Supplementary Table 1 Device parameters for the PSCs with different mol% of $(FEA)_2PbI_4$

Devices	J_{sc}	V_{oc}	*FF*	PCE
$MAPbI_3$	22.5±0.96	1.08±0.01	0.76±0.03	18.5±1.06
0.3 mol%	21.9±0.82	1.11±0.01	0.80±0.02	19.5±1.04
0.5 mol%	21.3±0.60	1.12±0.01	0.81±0.02	19.3±0.81
1 mol%	21.2±0.50	1.12±0.01	0.79±0.02	18.8±0.67
1.5 mol%	19.6±0.72	1.12±0.02	0.77±0.01	16.9±0.76
3 mol%	15.7±0.73	1.13±0.02	0.71±0.02	12.5±0.32
5 mol%	10.7±0.77	1.15±0.01	0.65±0.02	8.1±0.65

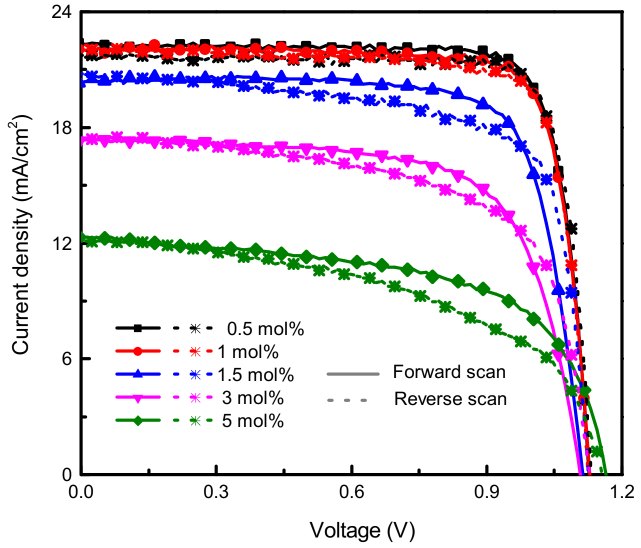

Supplementary Figure 1. ***J-V* curves of the devices containing different mol% of the $(FEA)_2PbI_4$ and $MAPbI_3$ components.** Note the data for the $MAPbI_3$ and 0.3 mol% $(FEA)_2PbI_4$ devices is shown in Figure 2a. The forward scans are solid lines with the reverse scans represented by dashed lines.

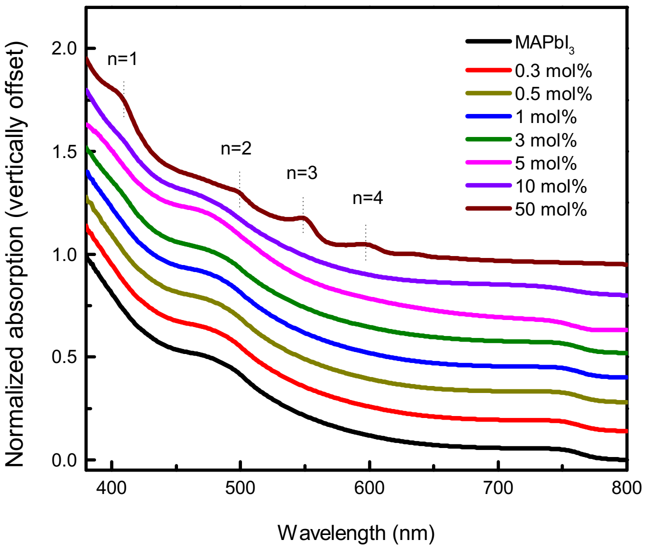

Supplementary Figure 2. **Normalized absorption spectra of perovskite films on glass substrates containing different mol% of $(FEA)_2PbI_4$.** The spectra are offset for clarity.

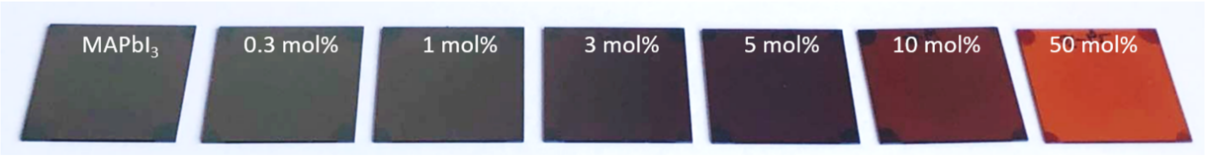

Supplementary Figure 3. **Pictures of perovskite films on glass substrates with different mol% of $(FEA)_2PbI_4$.**

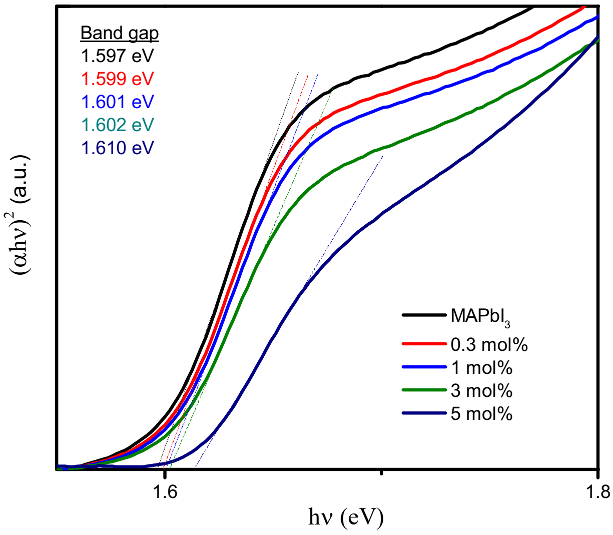

Supplementary Figure 4. **Tauc plots of perovskite films from UV-Vis spectra of Supplementary Figure 2.** $(\alpha h\nu)^2 = \beta(h\nu - E\text{g})$, where h is the Planck constant, ν is the photon frequency, Eg is the band gap energy, and β is a constant.

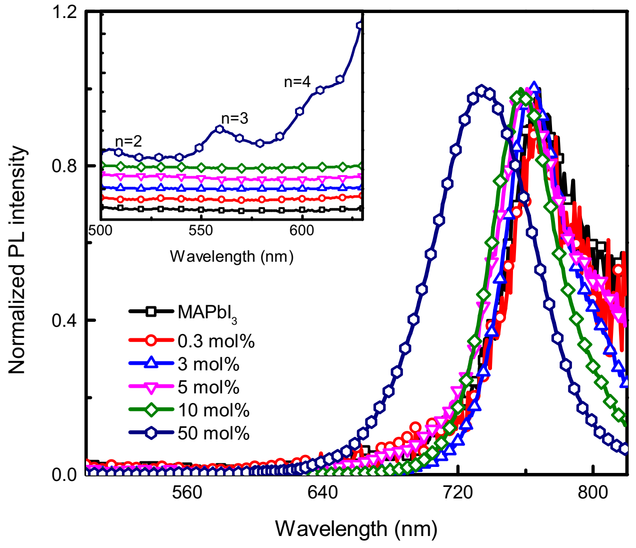

Supplementary Figure 5. **Normalized PL spectra of perovskite films containing different mol% of $(FEA)_2PbI_4$ on fused silica substrates (λ_{exc} = 450 nm).** PL = photoluminescence.

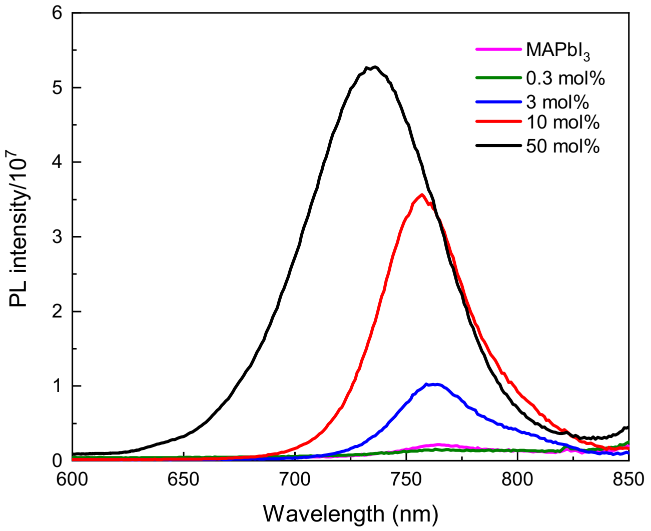

Supplementary Figure 6. **PL spectra of perovskite films containing different mol% of $(FEA)_2PbI_4$ on fused silica substrates (λ_{exc} = 450 nm).** PL = photoluminescence.

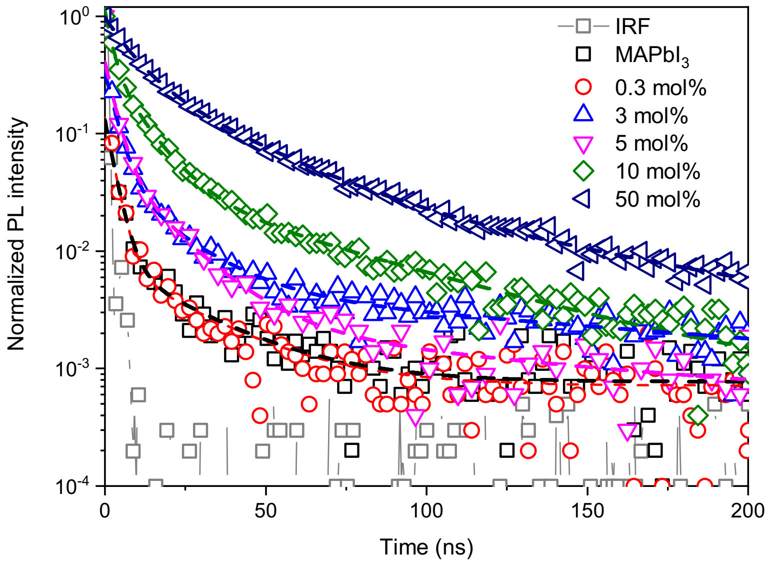

Supplementary Figure 7. **TRPL spectra of perovskite films containing different mol% of $(FEA)_2PbI_4$ on fused silica substrates.** PL = photoluminescence.

Supplementary Table 2 Fitting results from Supplementary Figure 7

Films	A_1	τ_1	A_2	τ_2	A_3	τ_3
$MAPbI_3$	96%	3.4 ns	4%	39.2 ns	\	\
0.3 mol%	96%	3.0 ns	4%	39.8 ns	\	\
3 mol%	87%	3.4 ns	11 %	16.8 ns	2%	183.3 ns
5 mol%	88%	4.1 ns	11%	19.5 ns	1%	146.1 ns
10 mol%	75%	3.9 ns	23%	15.5 ns	2%	93.9 ns
50 mol%	52%	7.0 ns	41%	22.9 ns	7%	80.1 ns

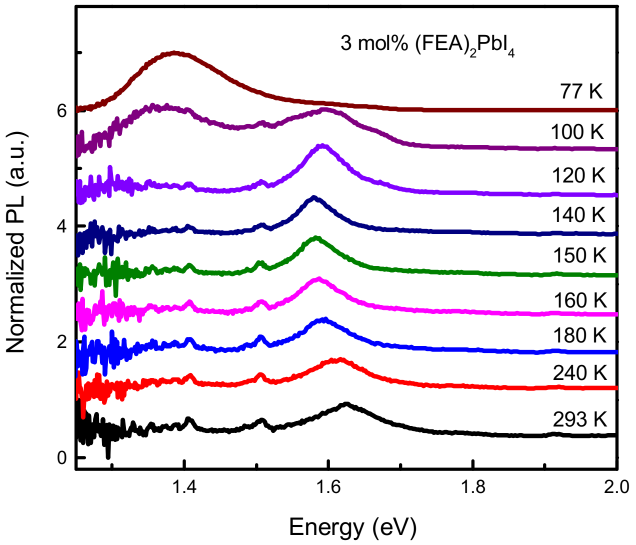

Supplementary Figure 8. **Temperature dependent PL spectra of the 3 mol% $(FEA)_2PbI_4$ film on glass substrate.** PL = photoluminescence.

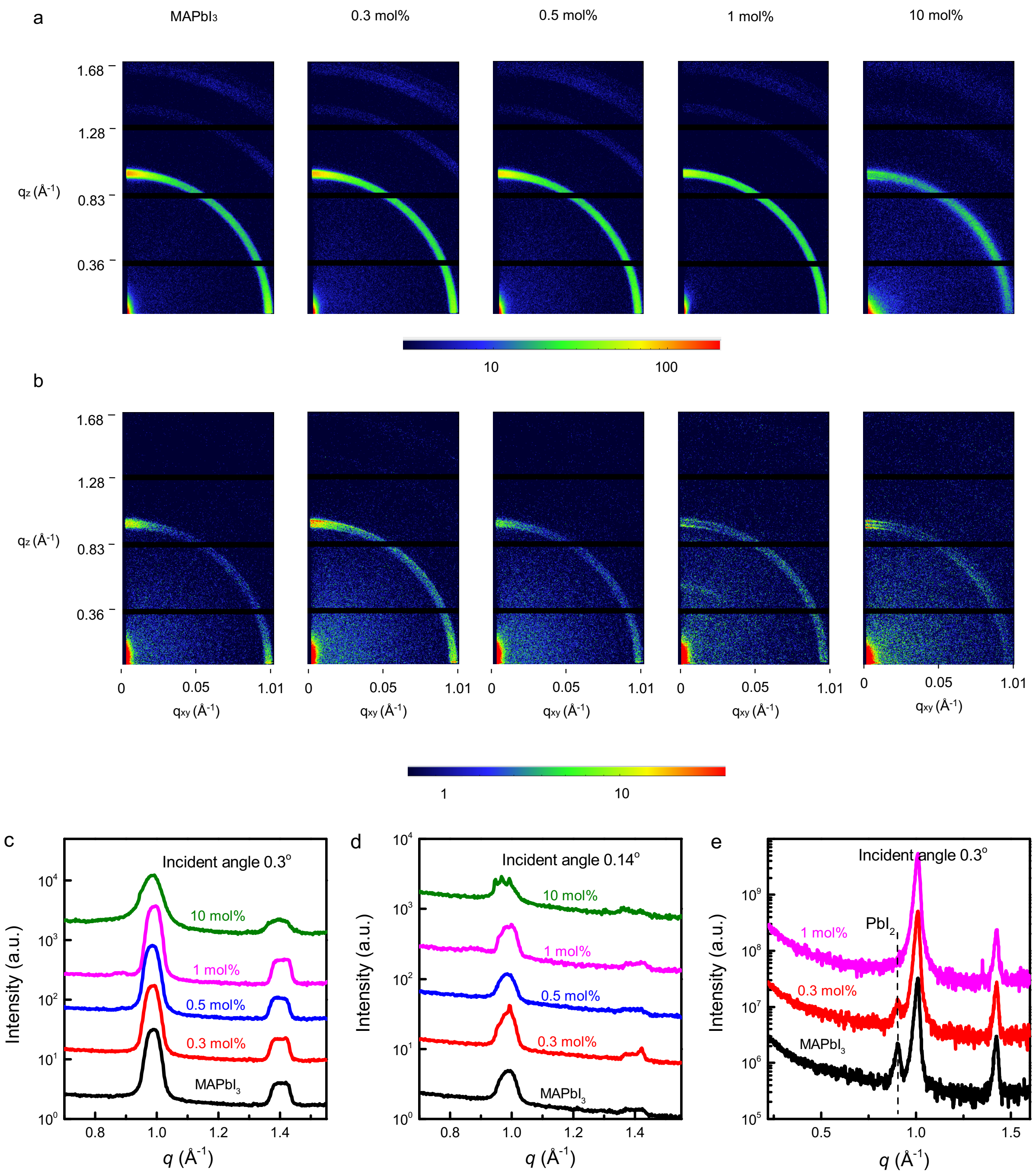

Supplementary Figure 9. **2D GI-WAXS measurements and out-of-plane high-resolution GI-XRD patterns of thin films.** 2D GI-WAXS measurements with incident beam of **a** 0.3° and **b** 0.14° for the $MAPbI_3$ and different mol% $(FEA)_2PbI_4$ films. Azimuthally averaged GI-WAXS profiles of the $MAPbI_3$ and 0.3 mol% $(FEA)_2PbI_4$ films collected at an incidence angle of **c** 0.3° and **d** 0.14°. **e** Out-of-plane high-resolution GI-XRD patterns of thin films collected at an incidence angle of 0.3° (Note the data have been offset for clarity). q = momentum transfer, q_z and q_{xy} = the components of the scattering vector (q) in the direction of the surface normal (z) and transverse to it (xy).

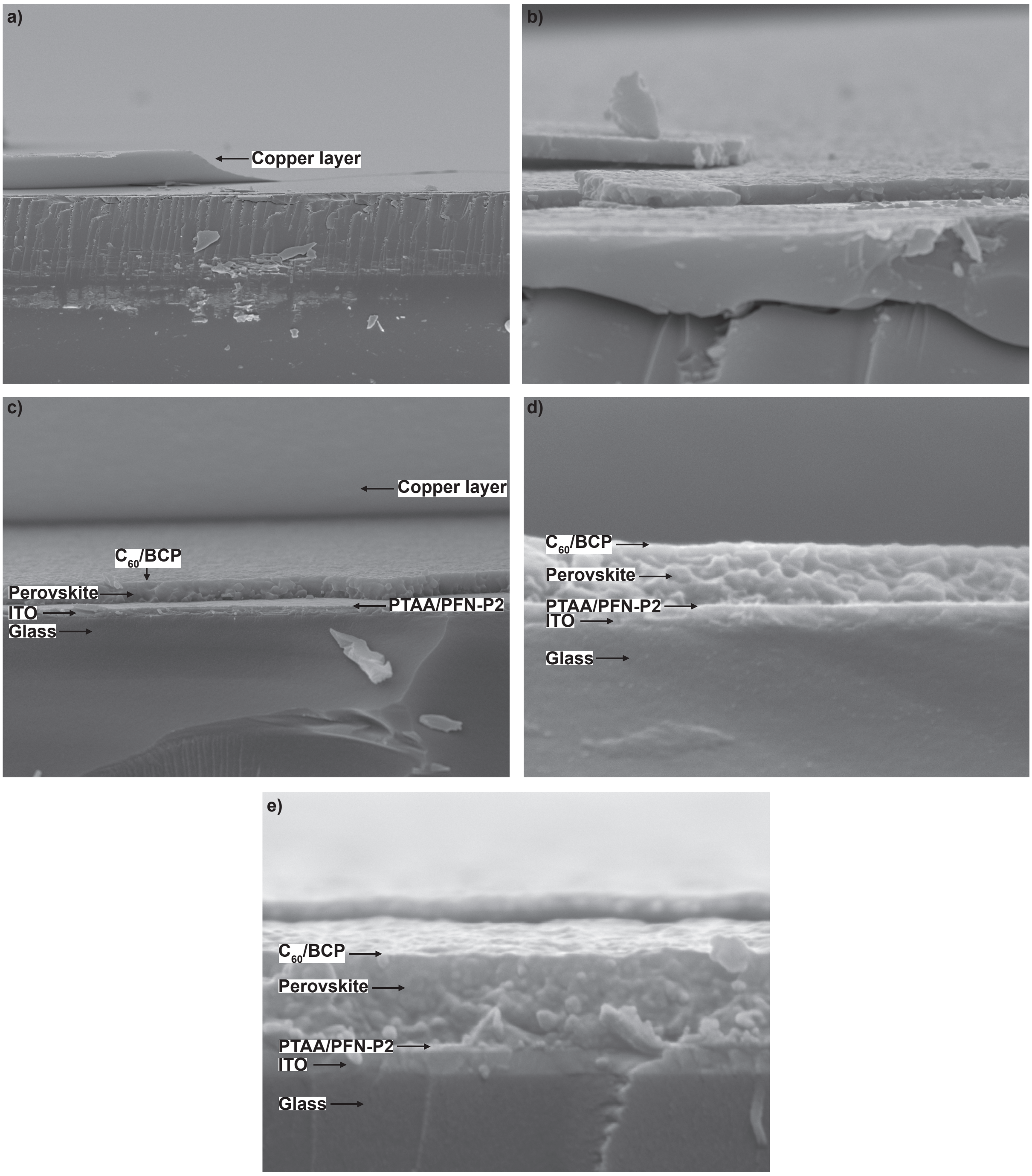

Supplementary Figure 10. **FESEM images of the full devices showing key layers and changes in the perovskite active layer structure. a** $MAPbI_3$-based device (×1000) showing the copper electrode flaking away from the surface caused by sample fracturing (note similar effect was seen for other devices). **b** $MAPbI_3$-based device (×15000). **c** 0.3 mol% FEAI-based device (×15,000). **d** 3.0 mol% FEAI-based device (× 60,000). **e** 10 mol% FEAI-based device (× 60,000). It should be noted that as the FEAI mol% increased the films became more prone to damage caused by the imaging process. The devices were scored with a glasscutter and snapped in half to expose cross-section of the device in a glovebox. The samples were then

mounted on a 90° angle for subsequent processes. Prior to SEM imaging, samples were sputter coated with platinum (10 nm) using a Q150TS sputter coater. The field-emission scanning electron microscope (FESEM) measurements were taken on a JEOL 7001F with accelerating voltage of 10 kEV and working distance of 9-10 mm.

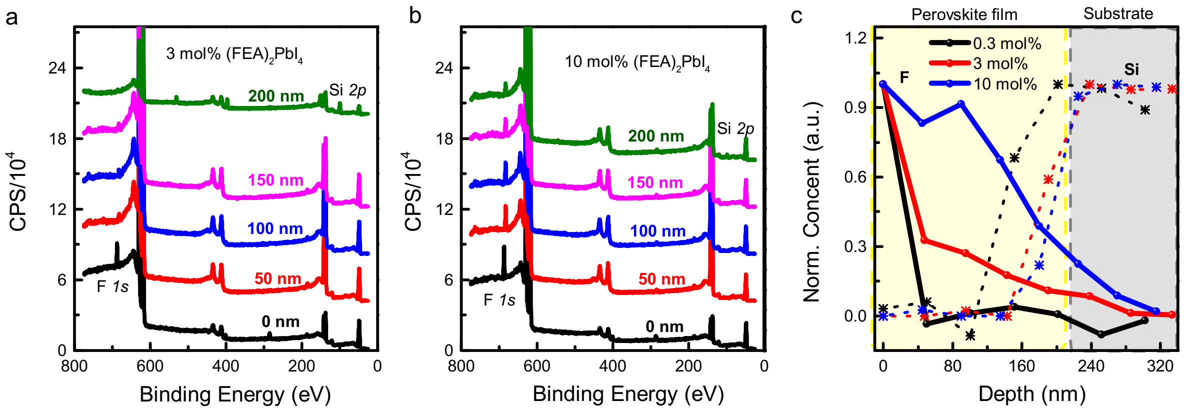

Supplementary Figure 11. **XPS depth profile of the films. a** 3 mol% $(FEA)_2PbI_4$. **b** 10 mol% $(FEA)_2PbI_4$. **c** Normalized concentration-depth profile of perovskite films with different $(FEA)_2PbI_4$ concentrations. CPS = counts per second.

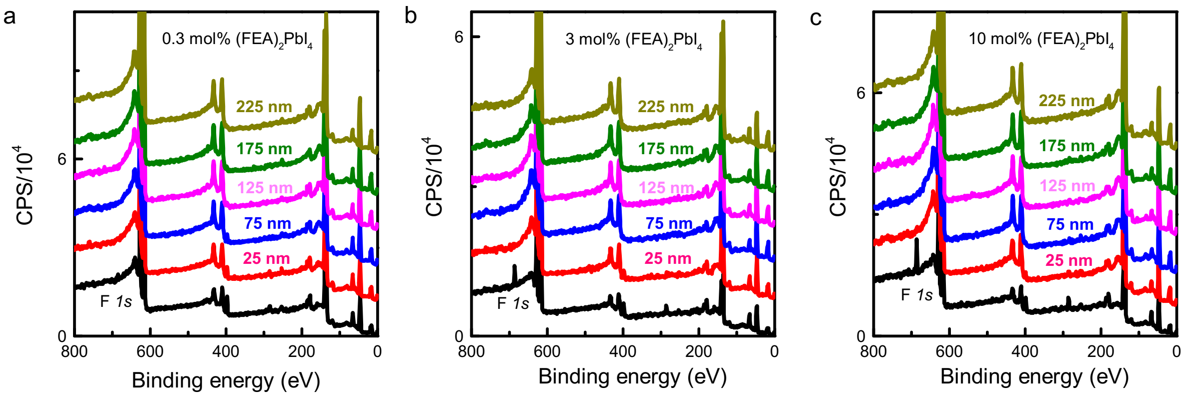

Supplementary Figure 12. **XPS depth profile of formamidinium lead triiodide films. a** 0.3 mol% $(FEA)_2PbI_4$. **b** 3 mol% $(FEA)_2PbI_4$. **c** 10 mol% $(FEA)_2PbI_4$. CPS = counts per second.

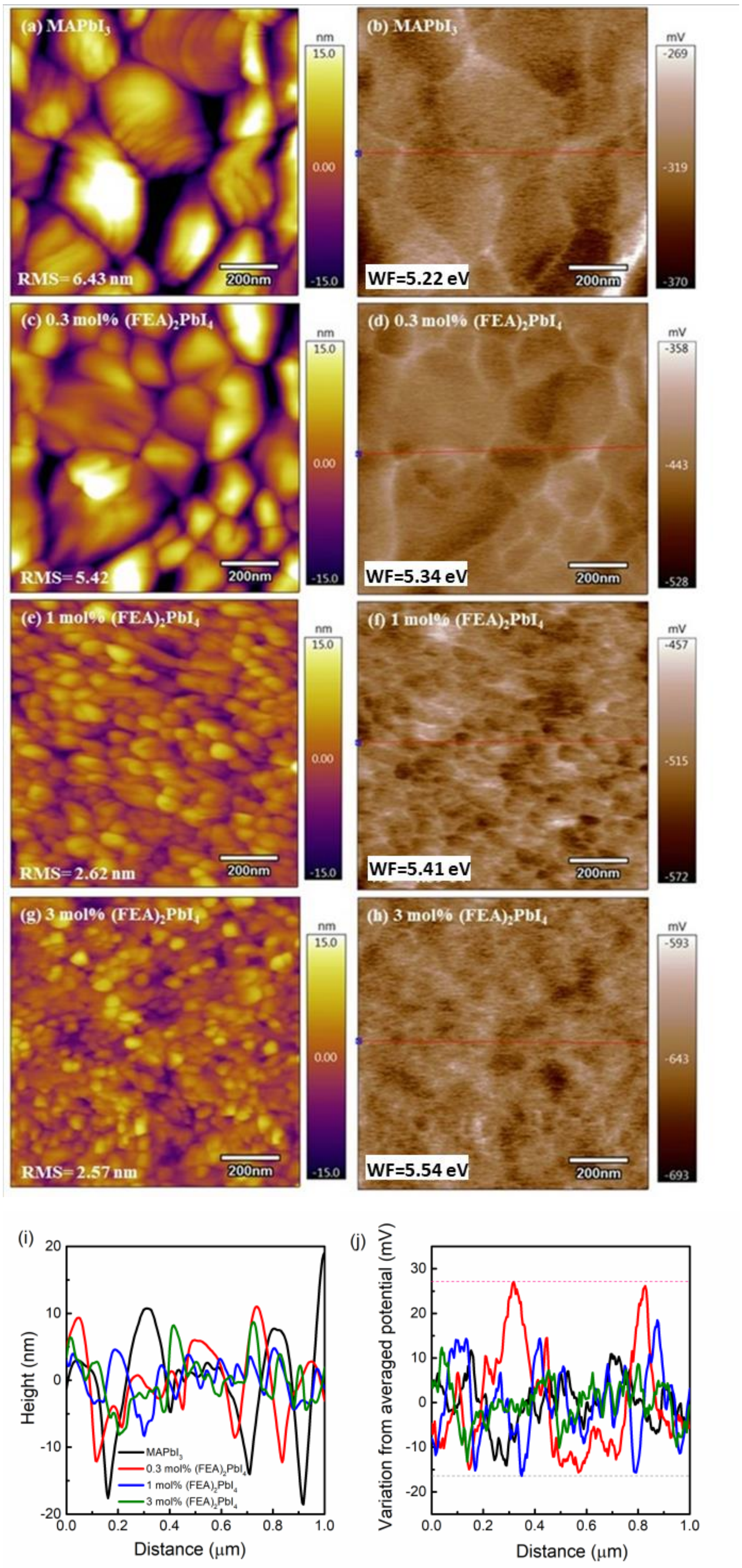

Supplementary Figure 13. **AFM and SKPM images. a** and **b** $MAPbI_3$ films. **c** and **d** 0.3 mol% $(FEA)_2PbI_4$. **e** and **f** 1 mol% $(FEA)_2PbI_4$. **g** and **h** 3 mol% $(FEA)_2PbI_4$. Cross-section profile of

topography **i** and CPD **j** from averages. RMS = root mean square roughness. WF = work function.

The AFM images show that with increasing $(FEA)_2PbI_4$ concentration the height difference between the grain surface and boundaries was decreased. It is also clear from comparison of the AFM and SKPM images that the surface potential difference of the films was highest at the grain boundaries (Supplementary Figure 13i and 13j). The average work functions of the $MAPbI_3$ and films with 0.3 mol%, 1 mol%, 3 mol% $(FEA)_2PbI_4$, were calculated from contact potential difference (CPD) and cantilever work function according to the equation $\varphi_{sample} = \varphi_{tip}\text{-}CPD$, and found to be 5.22 eV, 5.34 eV, 5.41 eV and 5.54 eV.

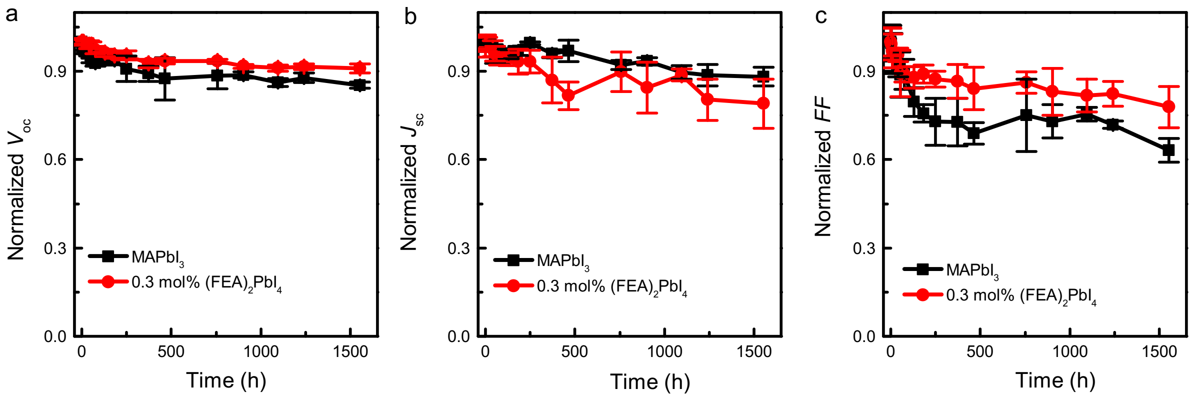

Supplementary Figure 14. **Thermal stability measurement photovoltaic parameters. a** V_{oc}. **b** J_{sc}. **c** *FF*.

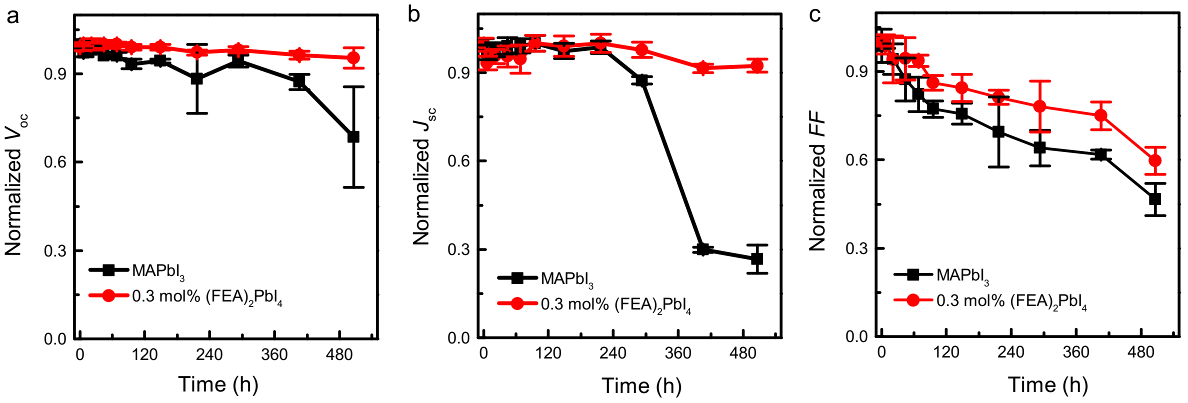

Supplementary Figure 15. **Humidity stability measurement photovoltaic parameters. a** V_{oc}. **b** J_{sc}. **c** *FF*.

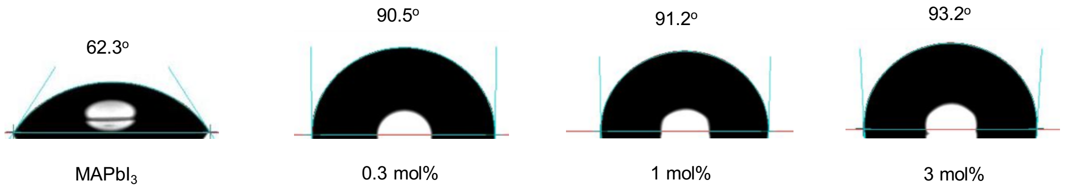

Supplementary Figure 16. **Images of water contact angles of perovskite films with 0.3 mol%, 1 mol%, and 3 mol% $(FEA)_2PbI_4$ on glass/ITO/PTAA/PFN-P2 substrates.**

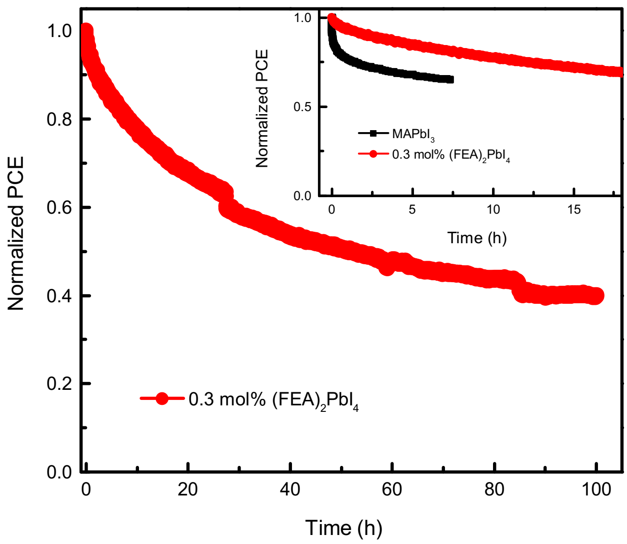

Supplementary Figure 17. **Normalised PCE for the $MAPbI_3$ and $(FEA)_2PbI_4$ lifetime test at maximum powerpoint.** The rapid burn in for the $MAPbI_3$ is similar to that previously reported. The initial PCEs of the $MAPbI_3$ and $(FEA)_2PbI_4$ devices were 19.7% and 17.4%, respectively. The stability of the solar cells was characterized using an in-house Labview J-V tracing program. A J-V scan (0-1.2 V) was performed every 60 seconds to determine the V_{mpp} while the P_{max} was recorded. An Abet AAB Sun 2000 Solar Simulator providing Air Mass 1.5 Global (AM 1.5G) illumination with power of 100 mW/cm^2 was used for the measurements. The measurements were undertaken in a glovebox (H_2O < 1 ppm and O_2 < 1 ppm) without encapsulation and with devices uncooled. PCE = power conversion efficiency.
