# Supplementary material for: Engineering fluorinated-cation containing inverted perovskite solar cells with an efficiency of >21% and improved stability towards humidity
Source: Nat Commun. 2021 Jan 4;12:52. doi: 10.1038/s41467-020-20272-3 (PMC7782759; doi:10.1038/s41467-020-20272-3)
Supplement: Supplementary file 2 — Reporting Summary [file 41467_2020_20272_MOESM2_ESM.pdf]

nature research

Corresponding author(s): Paul Burn

# Solar Cells Reporting Summary

Nature Research wishes to improve the reproducibility of the work that we publish. This form is intended for publication with all accepted papers reporting the characterization of photovoltaic devices and provides structure for consistency and transparency in reporting. Some list items might not apply to an individual manuscript, but all fields must be completed for clarity.

For further information on Nature Research policies, including our data availability policy, see Authors & Referees.

## Experimental design

**Please check: are the following details reported in the manuscript?**

1. Dimensions

| Item | Yes/No | Response |
|---|---|---|
| Area of the tested solar cells | ☒ Yes ☐ No | Methods |
| Method used to determine the device area | ☒ Yes ☐ No | Methods |

2. Current-voltage characterization

| Item | Yes/No | Response |
|---|---|---|
| Current density-voltage (J-V) plots in both forward and backward direction | ☒ Yes ☐ No | Main text (Figure 2a) and Figure S1 |
| Voltage scan conditions<br>*For instance: scan direction, speed, dwell times* | ☒ Yes ☐ No | Methods |
| Test environment<br>*For instance: characterization temperature, in air or in glove box* | ☒ Yes ☐ No | Methods, main text and supporting information |
| Protocol for preconditioning of the device before its characterization | ☐ Yes ☒ No | We did not precondition devices |
| Stability of the J-V characteristic<br>*Verified with time evolution of the maximum power point or with the photocurrent at maximum power point; see ref. 7 for details.* | ☒ Yes ☐ No | Main text and supporting information |

3. Hysteresis or any other unusual behaviour

| Item | Yes/No | Response |
|---|---|---|
| Description of the unusual behaviour observed during the characterization | ☒ Yes ☐ No | Main text |
| Related experimental data | ☒ Yes ☐ No | Main text and supporting information |

4. Efficiency

| Item | Yes/No | Response |
|---|---|---|
| External quantum efficiency (EQE) or incident photons to current efficiency (IPCE) | ☒ Yes ☐ No | Main text (e.g. Figure 2b) and methods |
| A comparison between the integrated response under the standard reference spectrum and the response measure under the simulator | ☒ Yes ☐ No | Methods and main text |
| For tandem solar cells, the bias illumination and bias voltage used for each subcell | ☐ Yes ☒ No | Tandems not studied |

5. Calibration

| Item | Yes/No | Response |
|---|---|---|
| Light source and reference cell or sensor used for the characterization | ☒ Yes ☐ No | Methods |
| Confirmation that the reference cell was calibrated and certified | ☒ Yes ☐ No | Methods |

Calculation of spectral mismatch between the reference cell and the devices under test

☒ Yes ☐ No — Methods

6. Mask/aperture

Size of the mask/aperture used during testing

☒ Yes ☐ No — Methods

Variation of the measured short-circuit current density with the mask/aperture area

☒ Yes ☐ No — Methods

7. Performance certification

Identity of the independent certification laboratory that confirmed the photovoltaic performance

☐ Yes ☒ No — Devices were not certified.

A copy of any certificate(s)
*Provide in Supplementary Information*

☐ Yes — *State where this information can be found in the text.*
☐ No — *Explain why this information is not reported/not relevant.*

8. Statistics

Number of solar cells tested

☒ Yes ☐ No — >30 and up to 50 in some cases, e.g., Figures 1 and 2

Statistical analysis of the device performance

☒ Yes ☐ No — Main text, e.g., Figures 1 and 2

9. Long-term stability analysis

Type of analysis, bias conditions and environmental conditions
*For instance: illumination type, temperature, atmosphere humidity, encapsulation method, preconditioning temperature*

☒ Yes ☐ No — Section on Solar Cell Stability and Supporting Information, Figure S17
